# Brain functional alterations in early stage of coal workers’ pneumoconiosis with alcoholism: insights from a resting-state fMRI investigation

**DOI:** 10.3389/fnins.2025.1610657

**Published:** 2025-06-16

**Authors:** Boting Xue, Gang Cao, Lingling Ren, Yuxiang Zhao, Xuecong Lv, Yong’ai Li, Hagiwara Akifumi, Yongbo Liu, Xiaowei Han

**Affiliations:** ^1^Department of Radiology, Peking University Lu’an Hospital, Affiliated of Shanxi Medical University, Changzhi, China; ^2^Department of Radiology, Changzhi People’s Hospital, Changzhi, China; ^3^Department of Radiology, Juntendo University School of Medicine, Tokyo, Japan; ^4^Department of Radiology, Graduate School of Medicine, University of Tokyo, Tokyo, Japan; ^5^Department of Radiology, The Quzhou Affiliated Hospital of Wenzhou Medical University, Quzhou People's Hospital, Quzhou, China

**Keywords:** coal worker’s pneumoconiosis, alcoholism, cognitive impairment, fMRI, amplitude of low-frequency fluctuation, functional connectivity

## Abstract

**Objectives:**

This study aimed to investigate resting-state functional magnetic resonance imaging (fMRI) changes in Coal Workers’ Pneumoconiosis (CWP) patients with alcoholism using Amplitude of Low-Frequency Fluctuation (ALFF) and Functional Connectivity (FC) analyses.

**Materials and methods:**

A total of 60 patients with stage I CWP and 30 healthy controls were prospectively enrolled. The CWP patients were further divided into drinking and non-drinking groups. Resting-state fMRI scans were performed for all three groups, and correlations between abnormal ALFF signals, FC changes, and clinical baseline data were analyzed.

**Results:**

Compared to the control group, both the CWP drinking and non-drinking groups exhibited increased ALFF signals in the left orbitofrontal cortex, left frontal pole, right intracalcarine cortex, and right precuneus cortex, as well as decreased ALFF signals in the bilateral temporal pole and left occipital fusiform gyrus. Functional connectivity analysis revealed increased connectivity from the right orbitofrontal cortex to the right pars triangularis of the inferior frontal gyrus and from the right precuneus cortex to the right lingual gyrus in both CWP groups. Conversely, decreased connectivity was observed in the left and right frontal poles. Additionally, a positive correlation was found between ALFF values in the left temporal pole and PaO2 levels in the CWP drinking group (r = 0.369, *p* = 0.038). In the CWP non-drinking group, functional connectivity between the right precuneus cortex and the right lingual gyrus showed a negative correlation with FVC (r = −0.442, *p* = 0.027).

**Conclusion:**

CWP patients with alcoholism exhibit abnormal brain regions and functional connectivity associated with spontaneous neural activity changes. Significant correlations between specific brain regions and clinical indicators were identified. These findings provide a foundation for understanding neuroimaging changes in CWP patients with alcoholism through fMRI.

## 1 Introduction

Coal Workers’ Pneumoconiosis (CWP) is an occupational disease caused by the long-term inhalation of mineral dust particles smaller than 5 μm in diameter. As of 2021, the number of coal dust workers is distributed in a double U shape, the incidence of CWP in China has been on the rise (Wang et al., 2023; Blackley et al., 2018), and about 20.6% of miners in the United States suffer from CWP. based on the GBZ 70–2015 national diagnostic criteria for occupational pneumoconiosis, CWP can be divided into three stages. The first stage is simple diffuse micronodules in both lungs, the second stage is aggregated micronodules, and the third stage is more complex and severe. Clinically, the irreversible pulmonary changes associated with CWP often result in chronic hypoxia, cyanosis, and progressive dyspnea. Chronic hypoxia has been linked to cognitive impairment, with studies suggesting that this relationship may stem from neurological damage. For instance, a study using a rat model of pneumoconiosis-associated cognitive impairment found that with the decrease of PaO2, chronic hypoxia led to diminished memory and learning abilities, likely due to cognitive and neurological abnormalities (Lv and Niu, 2012). On the other hand, Alcoholism is a known risk factor for alcohol-related liver disease, alcohol-induced cardiomyopathy and liver cancer (Danpanichkul et al., 2024), In addition, alcoholism has been shown to have impact on Alzheimer’s disease (AD), which is associated with structural and functional brain changes. It impairs attention, memory, and emotional and perceptual functions, contributing significantly to cognitive dysfunction (Koch et al., 2019; Dai et al., 2023). Alcoholism, therefore, plays a critical role in cognitive impairment (Selim et al., 2024; Pagni et al., 2024). Alcoholism is prevalent among CWP patients; however, studies investigating cognitive function changes in CWP patients with alcoholism remain scarce.

CWP has been shown to cause cognitive impairment through chronic hypoxia resulting from hypoxemia and chronic microvascular injury (Imaizumi et al., 2014), while alcoholism can directly affect the brain or lead to neurotrophic factor deficiencies, resulting in cognitive changes and even dementia (Sabia et al., 2014). According to the DSM-V, alcohol dependence can cause mild cognitive impairment (MCI), an intermediate stage between normal aging and dementia, characterized by cognitive decline without significant impact on daily activities, which may progress to more severe stages (Cong et al., 2023). Although the mechanisms of CWP and alcoholism differ, both can induce MCI, leading to varying degrees of cognitive and executive function impairment. Neuroimaging studies using functional Magnetic Resonance Imaging (fMRI), particularly Blood Oxygenation Level Dependent (BOLD) fMRI, have revealed changes associated with MCI (Zhe et al., 2023). For example, ALFF studies in neurodegeneration have shown reduced ALFF signals in the right medial paracentral cingulate gyrus, bilateral inferior cerebellar lobes, and bilateral precuneus cortex (Yang et al., 2018), while chronic smokers have shown different Functional Connectivity in the dorsal attention network (Zhang et al., 2017; Weidler et al., 2024). Similarly, altered ALFF signals have been reported in patients with co-morbid alcohol dependence and depression (Sun et al., 2023). These findings suggest that both pneumoconiosis-induced hypoxia and alcoholism can disrupt spontaneous neural activity in the brain, contributing to abnormal cognitive function (Marshall et al., 2015).

In this study, we hypothesize that CWP combined with alcoholism may exhibit abnormal spontaneous brain activity compared to CWP without alcohol consumption. To test this, we propose using Amplitude of Low-Frequency Fluctuation (ALFF) and seed-based functional connectivity (FC) analyses to investigate alterations in spontaneous neural activity in the brains of CWP patients with alcoholism and explore the relationship between these alterations and clinically relevant cognitive indices. Specifically, we will first compare differences in ALFF and FC indices among three groups: the CWP drinking group, the CWP non-drinking group, and the healthy control group, to identify abnormal functional brain regions. Secondly, we will analyze the correlation between imaging indices of these brain regions and clinical cognitive assessment indices. This study aims to provide insights into the abnormal neuropathological changes associated with CWP and alcoholism from an imaging perspective.

## 2 Materials and methods

### 2.1 Participants

In this study, 60 male stage I CWP patients who had worked in the coal mining industry and were diagnosed based on the GBZ 70–2015 national diagnostic criteria for occupational pneumoconiosis between 2023 and 2024 were prospectively enrolled. During the same period, 30 healthy controls were matched for age, gender, and education level. The Personal Drinking Habits Questionnaire (PDHQ) was used to collect information on general drinking habits of CWP patients, including drinking years and alcohol consumption per tael. Based on this scale, we divided CWP patients into two groups: those with alcoholism (male patients with a history of drinking behavior of >1 drink and <14 drinks per week (National Institute on Alcohol Abuse and Alcoholism, 2004), drinking group) and those with no history of drinking (non-drinking group). A total of 90 participants were included: 32 in the drinking group, 28 in the non-drinking group, and 30 in the control group.

The inclusion criteria for CWP patients were: (1) The patients who were strictly diagnosed as stage 1 of CWP showed diffuse micronodules in both lungs on high kV X-ray and currently active or retired employees, (2) age ≥50 years, (3) right-handed, (4) education level ≥6 years, and (5) signed informed consent to complete both the cognitive scale test and MRI scanning. The inclusion criteria for CWP patients with alcoholism were: (1) male patients with a history of drinking behavior of >1 drink and <14 drinks per week, (2) diagnosed as CWP stage 1, (3) age ≥50 years, (4) right-handed, (5) education level ≥6 years. The inclusion criteria for the control group were: (1) age ≥50 years, (2) right-handed, (3) education level ≥6 years, and (4) signed informed consent to complete cranial MRI scanning. The exclusion criteria for all participants were: (1) organic cranial brain lesions, (2) psychoneurological disorders, (3) pneumoconiosis combined with COPD or obstructive sleep apnea-hypopnea syndrome, (4) a history of alcoholism, alcohol use disorder, or sedative-hypnotic drug abuse, and (5) contraindications to MRI examination, such as artificial metal implants or claustrophobia. The study protocol was approved by the Medical Ethics Committee of our hospital (approval number: 2022009) and was conducted in strict compliance with the principles of the Declaration of Helsinki. All participants provided written informed consent for inclusion in the study.

### 2.2 Neurocognitive measures and laboratory tests

One hour prior to undergoing an MRI scan, the subjects completed a series of cognitive and psychological assessments, including the Mini-Mental State Examination (MMSE), the Montreal Cognitive Assessment Scale-Basic (MoCA-B) Chinese version, the Subjective Cognitive Decline Assessment (SCD-Q), the Hamilton Anxiety Scale (HAMA), the Hamilton Depression Scale (HAMD), the Geriatric Depression Scale (GDS), and the Clock Drawing Test (CDT). Both the MMSE and MoCA scales were scored out of 30, with higher scores indicating better overall cognitive function. Additionally, clinical data were collected, including partial pressure of oxygen (PaO2) and pulmonary function test results, such as forced expiratory volume in 1 sec (FEV1), forced vital capacity (FVC), and the one-second rate (FEV1/FVC).

### 2.3 MR imaging data acquisition

Imaging data were collected using a 3.0 T Prisma (Siemens, Germany) scanner equipped with a 64-channel head-specific coil in the imaging department of our Hospital. Conventional scanning techniques were applied for T1-weighted imaging (T1WI), T2-weighted imaging (T2WI), and T2-FLAIR sequences, while BOLD sequences were acquired using gradient echo planar imaging with the following parameters: 36 slices, repetition time (TR) 2,190 ms, echo time (TE) 30 ms, slice thickness 3.0 mm, flip angle 90°, field of view (FOV) 200 mm × 200 mm, in-plane resolution 64 × 64, 180 repetitions, and an acquisition time of 6 min. Structural T1 images were obtained using 3D T1-weighted scrambled-phase gradient echo sequences (sagittal orientation) with the following parameters: FOV 256 × 256 mm, in-plane resolution 256 × 256, 176 slices, slice thickness 1 mm, TR 1,900 ms, TE 2.48 ms, inversion time 900 ms, flip angle 9°, and slice gap 0 mm. During the scanning process, subjects were instructed to keep their eyes closed, stay awake, and avoid significant head movements. All imaging data were thoroughly reviewed and verified by two experienced radiologists to ensure quality and accuracy.

### 2.4 Image data preprocessing

The acquired images were preprocessed using MATLAB R2023a^1^ with the Statistical Parametric Mapping software (SPM12, www.fil.ion.ucl.ac.uk/spm/) and the Data Processing Assistant for Resting-State Functional Magnetic Resonance Imaging toolbox (DPARSF v8.1, http://www.restfmri.net/forum/DPARSF) to process both structural and functional images (Yan et al., 2016). Functional image preprocessing included eliminating the first 10 time points, slice timing correction, excluding subjects with head motion >2.0° rotation or translation, aligning structural and functional images, spatial normalization, covariate regression (Friston 24 model of head motion parameters, CSF signal, and white matter signal), spatial smoothing with isotropic Gaussian kernels (FWHM = 4 mm), and band-pass filtering (0.01–0.1 Hz) to remove physiological high-frequency noise in BOLD images. During quality control, three subjects from the CWP group and one from the control group were excluded due to excessive head motion or image artifacts. Ultimately, 32 subjects in the drinking group, 25 in the non-drinking group, and 29 in the healthy control group met the inclusion criteria and were included in the analysis.

### 2.5 ALFF analysis

Due to the design involving multiple between-group samples, a one-way analysis of variance (ANOVA) based on DPABI with post-hoc comparisons was employed to analyze the szALFF results obtained after preprocessing. Gender, age, smoking history (Nicotine could affects spontaneous neural activity), and the mean FD Jenkinson coefficient were included as main covariates. The results were corrected using Gaussian random field (GRF) theory with a cluster definition threshold of *p* = 0.005 (two-tailed), corresponding to a cluster-level threshold of *p* = 0.05, to strictly control the false positive rate and identify abnormal brain regions. Whole-brain mean ALFF values of the abnormal brain regions were extracted using the DPABI-based ROI signal extraction procedure. Spearman correlation analysis was then conducted to examine the relationship between the mean ALFF values of the abnormal brain regions and clinical cognitive correlates.

### 2.6 FC analysis

Using the DPABI Viewer, the signals of ALFF abnormal brain regions were defined as regions of interest (ROIs). Gender, age, smoking history, and the mean FD Jenkinson coefficient were included as main covariates, and the results were corrected using Gaussian random field (GRF) theory. Clustering was defined by a threshold of *p* = 0.005 (two-tailed), corresponding to a cluster-level threshold of p = 0.05, to strictly control the false positive rate and display the FC of each ROI with the whole brain. FC brain maps were generated using BrainNet Viewer v1.7.^2^ The DPABI-based ROI signal extraction procedure was used to extract whole-brain mean FC values for the ROIs of abnormal brain regions. Spearman correlation analysis was then performed to assess the relationship between the mean FC values of abnormal brain regions and clinical cognitive correlates.

### 2.7 Statistical analysis

IBM SPSS Statistics 26.0 (SPSS Inc. Chicago, IL) was applied to test the age, education, and cognitive scores etc. Measures that met normal distribution as well as variance chi-square were expressed as x̄ ± s, independent samples t-tests were performed between two groups, and ANOVA analyses were performed between multiple groups. Measures that did not conform to normal distribution and variance chi-square or only one conformed were expressed as median (upper and lower quartiles), Mann–Whitney U test was performed between the two groups, and Kruskal-Wallis non-parametric test was performed between multiple groups. *p* < 0.05 means the difference was statistically significant.

## 3 Results

### 3.1 Demographic and clinical profiles

All subjects enrolled in this study were male. Significant differences were observed between the CWP drinking group and the CWP non-drinking group in terms of HAMA, HAMD, and GDS scores (*p* < 0.05), with the CWP drinking group showing higher scores for SCD-Q, HAMA, HAMD, and GDS compared to the non-drinking group. However, no significant differences (*p* > 0.05) were found between the two groups in clinical and cognitive scale indicators such as age, education, years of coal mining, years of drinking, alcohol consumption per tael, ALT, FVC, FEV1, FEV1/FVC, PaO2, MMSE, MoCA, SCD-Q, and CDT. Notably, MoCA scores and lung function indices were lower in the non-drinking group compared to the drinking group (Table 1).

**Table 1:** Clinical profile of baseline conditions and scale scores for the coal workers’ pneumoconiosis group and the control group.

Characteristic	Drinking groups (*n* = 32)	Nondrinking groups (*n* = 25)	Controls (*n* = 29)	Statistic value	*p* value
Age	57.96 ± 5.029	57.88 ± 6.333	58.38 ± 4.459	H = 0.758	0.684
Education	12 (9,12)	9 (6.25,12)	9 (6,12)	H = 1.835	0.399
Working years	28 (20,34)	28 (20.25,31)		Z = −0.298	0.766
Drinking years	21.16 ± 12.216				
AC/tael	2 (2,4.5)				
ALT	19.90 (13.70,27.45)	20.05 (15.78,30.05)		Z = −0.434	0.664
FVC	84 (79.50,95.50)	80 (80,88.75)		Z = −0.946	0.344
FEV1	79.68 ± 12.165	77.47 ± 15.643		t = 0.582	0.563
FEV1/FVC	91 (82.50,95)	87 (78.25,92)		Z = −1.248	0.212
PaO2	97 (96,98.50)	97 (96,98)		Z = −0.662	0.508
MMSE	28 (26,29)	28 (27,29)		Z = −1.344	0.179
MoCA	22.92 ± 3.081	22.66 ± 2.858		t = 0.334	0.740
SCD-Q	6 (4.5,7.5)	5.5 (4,6.5)		Z = −1.517	0.129
HAMD	5.5 (3.25,7)	2 (1.5,7)		Z = −2.219	0.027
HAMA	6 (5.25,9)	4 (3,7)		Z = −2.420	0.016
GDS	4 (2,6)	3 (1,4)		Z = −1.980	0.048
CDT	22 (12.25,27.75)	24 (15,27)		Z = −0.291	0.771

AC/tael, Alcohol Consumption per tael. MMSE, Minimum Mental State Examination, MoCA, Montreal Cognitive Assessment, SCD-Q, Subjective Cognitive Decline questionnaire, HAMD, Hamilton Depression Scale, HAMA, Hamilton Anxiety Scale, GDS, geriatric depression scale, CDT, Clock Drawing Test.

### 3.2 ALFF comparison

**Figure 1:**
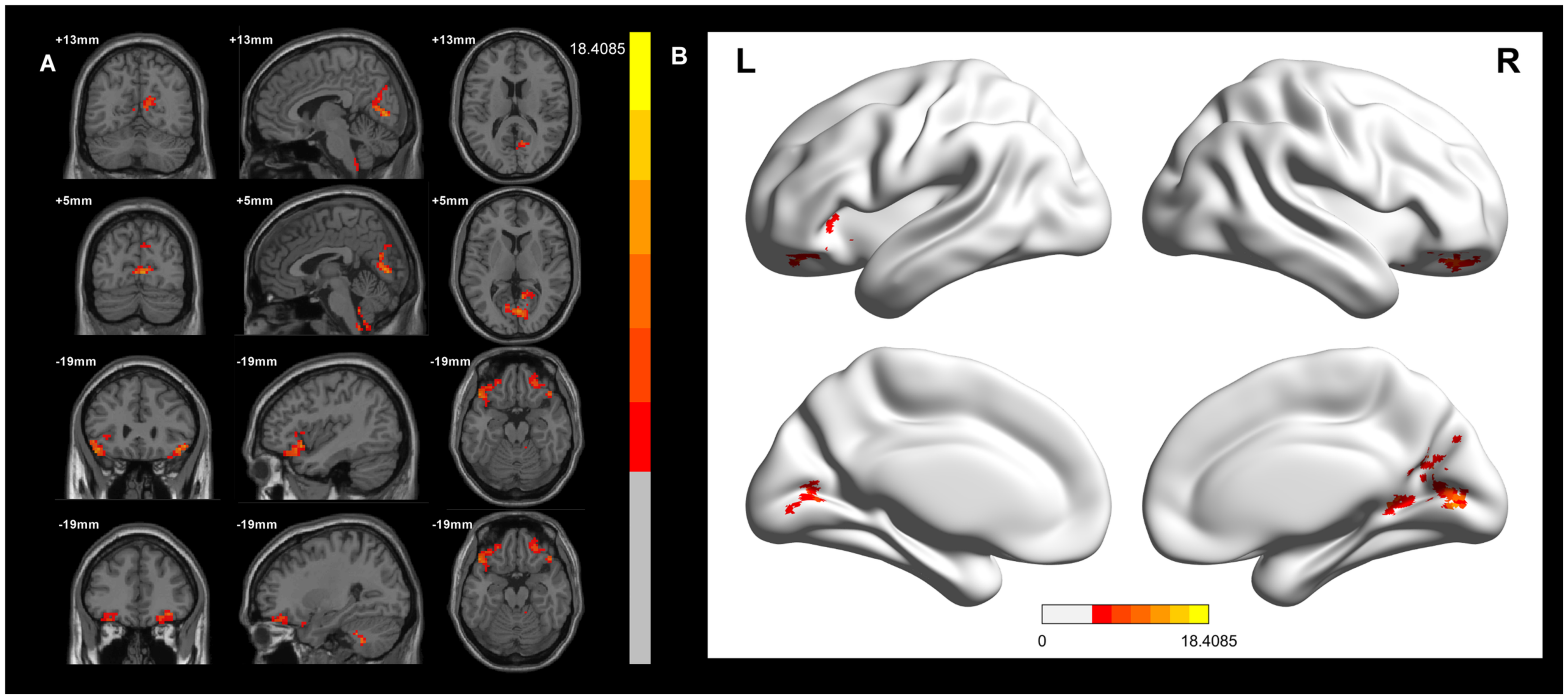
ALFF signal differences and 3D brain images in the CWP drinking group, CWP non-drinking group and control group. In the three-group analysis. **(A)** ALFF signals were significantly higher in the right Precuneus Cortex, right Intracalcarine Cortex, Bilateral Orbitofrontal Cortex (two-tailed, voxel *p* < 0.005, GRF, cluster level *p* < 0.05). **(B)** 3D brain images showed higher ALFF signals in the right Precuneus Cortex, right Intracalcarine Cortex, Bilateral Orbitofrontal Cortex (*F* values<18.4085). Colorbar represent *F*-values, ALFF: amplitude of low-frequency fluctuations; L: left, R: right.

In the voxel-based one-way ANOVA analysis, ALFF signals were significantly increased in the right Precuneus Cortex, right Intracalcarine Cortex, and Bilateral Orbitofrontal Cortex. among the three groups: CWP drinkers, CWP non-drinkers, and controls (GRF, voxel *p* < 0.005, cluster *p* < 0.05, Figure 1). Some of these brain regions were associated with the default mode network (DMN). Table 2 provides the cluster information and MNI coordinates of the brain regions with significant differences.

**Table 2:** Clustered information on brain regions of differences between the coal workers’ pneumoconiosis group and the control group.

Cluster	Brain areas (HOA)	Voxel size	Peak coordinate			*t*-value
			X	Y	Z	
1	Frontal Orbital Cortex (L)	41	−45	24	−15	14.706
	Frontal Pole (L)	10	−39	35	−19	
2	Intracalcarine Cortex (R)	25	3	−75	6	13.975
	Precuneus Cortex (R)	10	13	−61	17	

HOA, Harvard-Oxford Cortical Structural Atlas. L: left; R: right. 𝑋, 𝑌, and 𝑍: coordinates of primary peak locations in the MNI space. A positive *t* value indicates increased ALFF, and a negative *t* value indicates decreased ALFF.

**Figure 2:**
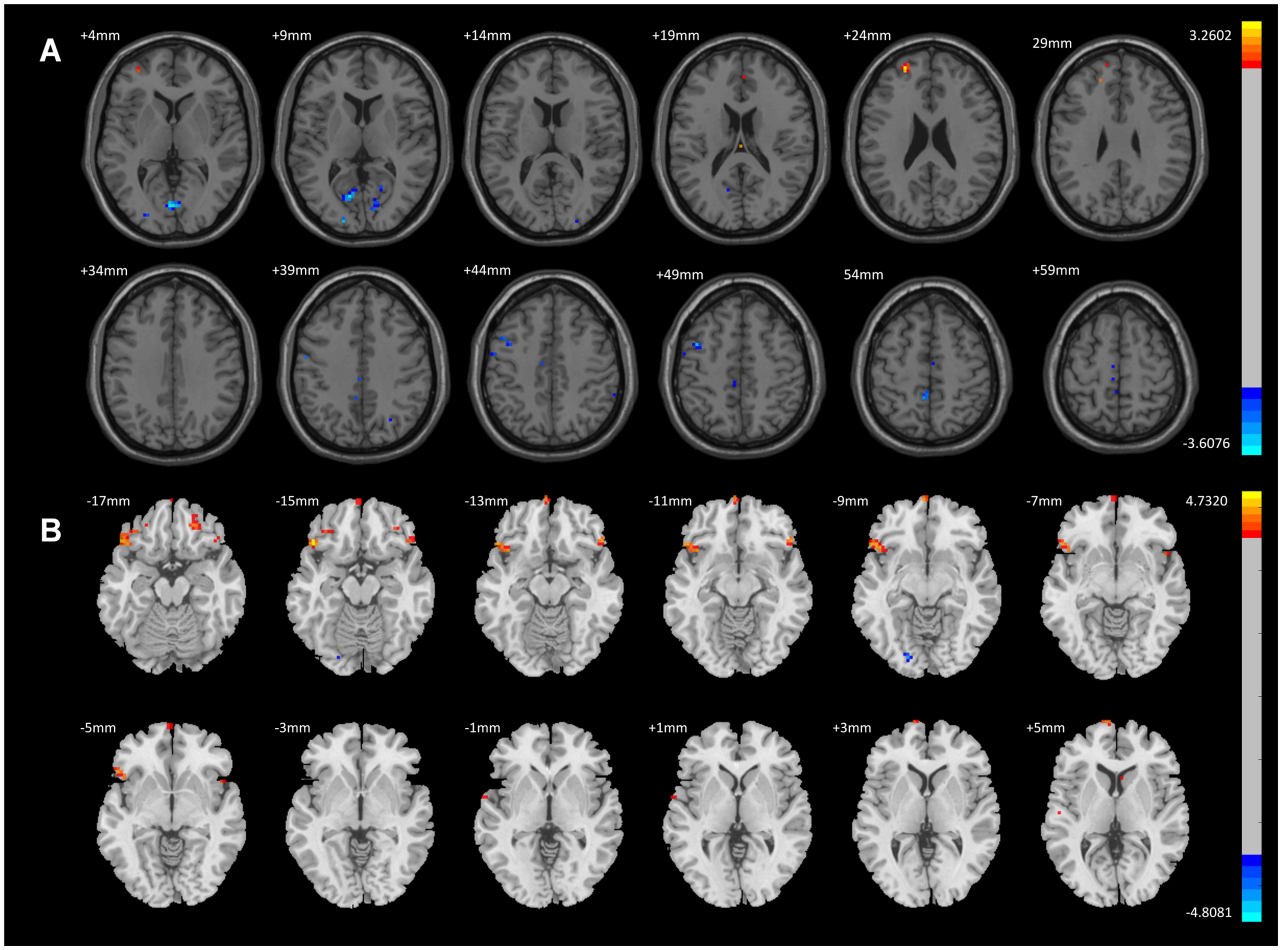
ALFF Differential Brain Regions in CWP Drinking Group and CWP Non-Drinking Group, CWP drinking and control group. **(A)** Compared with the CWP Non-Drinking Group, ALFF signals were significantly increased in the left Frontal Pole of the drinking group; ALFF signals were decreased in the left Postcentral Gyrus, Intracalcarine Cortex, Middle Frontal Gyrus, and Lingual Gyrus (two-tailed, voxel *p* < 0.001, GRF, cluster level *p* < 0.05). **(B)** Compared with the control group, ALFF signal was increased in the bilateral Orbitofrontal Cortex, left Frontal Pole, and decreased in the left Occipital Fusiform Gyrus in the drinking group (two-tailed, voxel p < 0.001, GRF, cluster level *p* < 0.05). GRF: Gaussian random field, Color bars represent t-values.

When comparing the CWP drinking group to the CWP non-drinking group, the left Frontal Pole showed significantly increased ALFF signals, while decreased ALFF signals were observed in the left Posterior Central Gyrus, left Intracalcarine Cortex, left Middle Frontal Gyrus, and Lingual Gyrus (GRF, voxel p < 0.005, cluster *p* < 0.05, Figure 2A).

Compared to the control group, the CWP drinking group exhibited increased ALFF signals in the bilateral Orbitofrontal Cortex and left Frontal Pole, and decreased ALFF signals in the left Occipital Fusiform Gyrus (GRF, voxel *p* < 0.001, cluster *p* < 0.05, Figure 2B).

**Figure 3:**
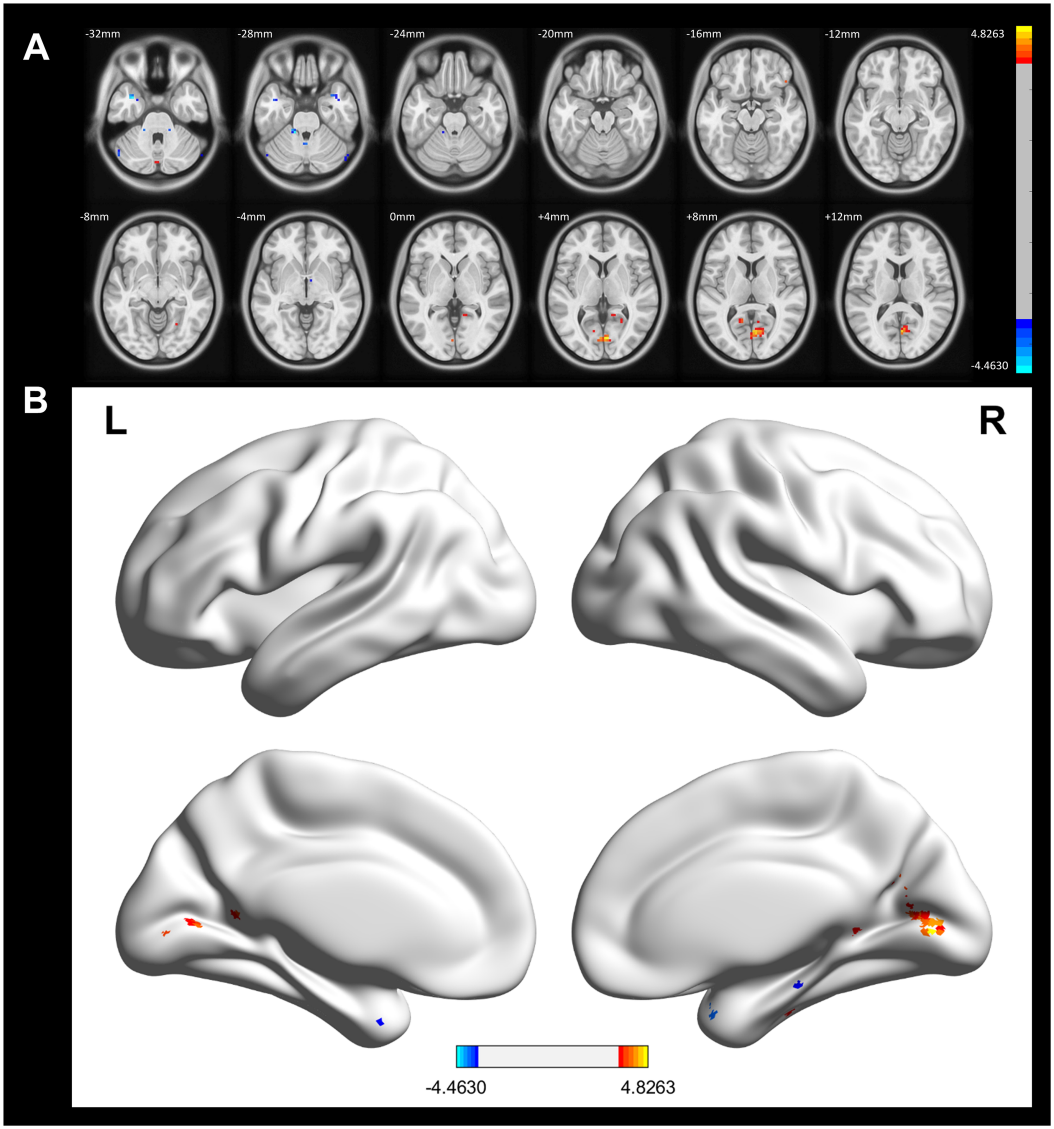
ALFF difference brain regions and 3D brain images of CWP non-drinking group and control group. **(A)** Compared with the control group, the non-drinking group was seen to have increased ALFF signals in the right Intracalcarine Cortex and Precuneus Cortex, and decreased signals in the bilateral Temporal Poles (two-tailed, voxel *p* < 0.001, GRF, cluster level *p* < 0.05). **(B)** 3D brain images showed increased ALFF signals in the right Intracalcarine Cortex and Precuneus Cortex, and decreased signals in the bilateral Temporal Poles (−4.4630 < F values<4.8263) Color bars represent *t*-values, L: left, R: right.

In contrast, compared to the control group, the CWP non-drinking group showed increased ALFF signals in the right Intracalcarine Cortex and Precuneus Cortex, and decreased ALFF signals in the bilateral Temporal Poles (GRF, voxel *p* < 0.001, cluster *p* < 0.05, Figure 3).

### 3.3 FC comparison

**Figure 4:**
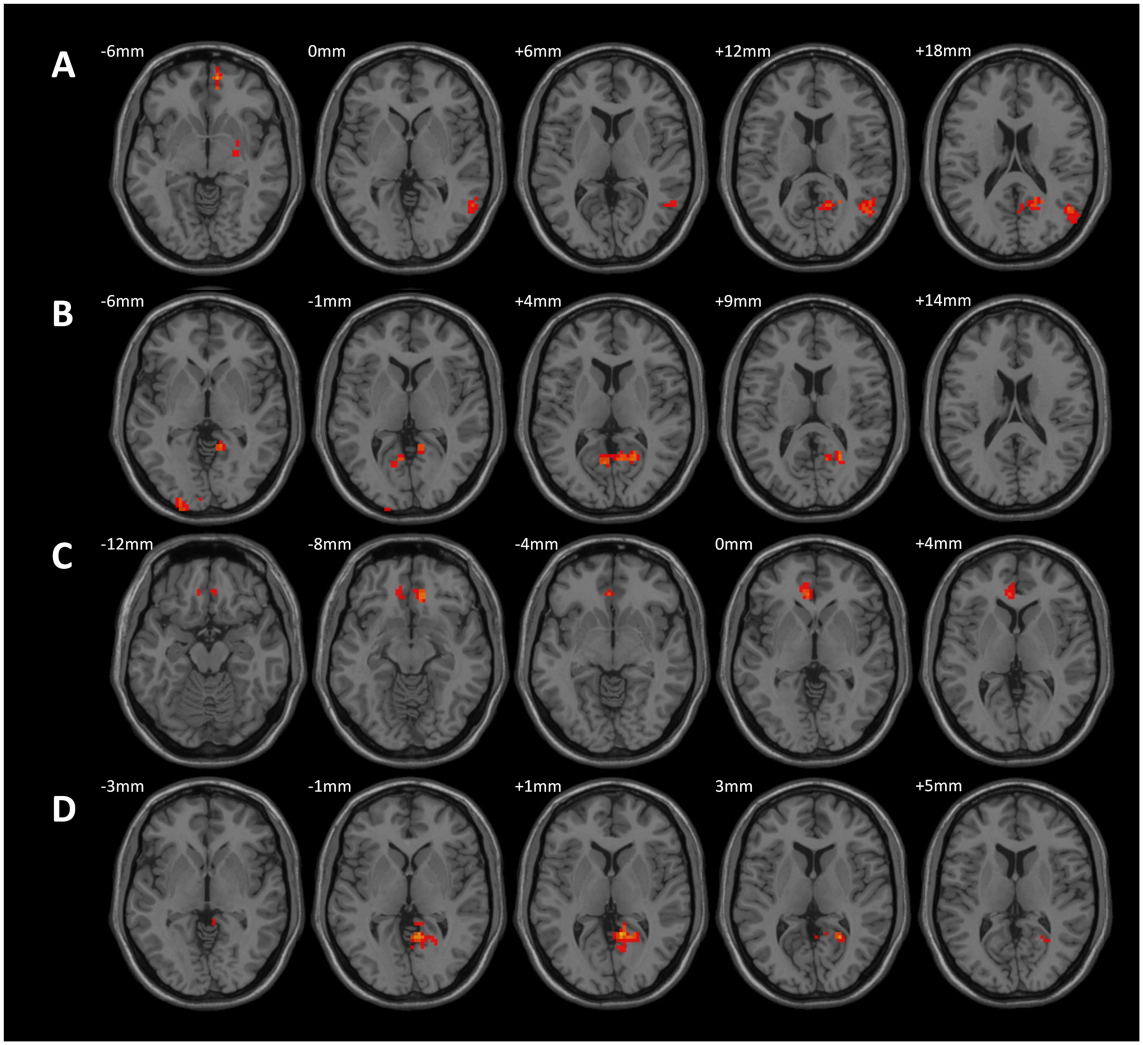
Differences in functional connectivity in the CWP drinking group, CWP non-drinking group, and control group. **(A)** Increased functional connectivity in the left Orbitofrontal Cortex to the right Frontal Pole, right Lateral Occipital Cortex (top), and right Precuneus Cortex. **(B)** Increased functional connectivity in the left Frontal Pole to bilateral Precuneus Cortex, left Occipital Fusiform Gyrus, right Inferior Frontal Gyrus, and left Occipital Gyrus. **(C)**: Increased functional connectivity in the right Intracalcarine Cortex to bilateral Medial Frontal Cortex and left Cingulate Gyrus. **(D)**: Increased functional connectivity in right Precuneus Cortex and right Lingual Gyrus (GRF, voxel *p* < 0.005, cluster *p* < 0.05). l: left side, r: right side.

In a voxel-based one-way ANOVA analysis, the connectivity of the left Orbitofrontal Cortex to the right Frontal Pole, right Lateral Occipital Cortex (superior), and right Precuneus Cortex was significantly increased across the three groups: the CWP drinking group, the CWP non-drinking group, and the control group. The left Frontal Pole showed increased connectivity to the bilateral Precuneus Cortex, left Occipital Fusiform Gyrus, right Inferior Frontal Gyrus, and left Occipital Lobe. Additionally, the right Intracalcarine Cortex exhibited increased connectivity to the bilateral Medial Frontal Cortex and the left Cingulate Gyrus. The connectivity of the right Precuneus Cortex to the right Lingual Gyrus was also increased (GRF, voxel *p* < 0.005, cluster *p* < 0.05, Figure 4).

**Figure 5:**
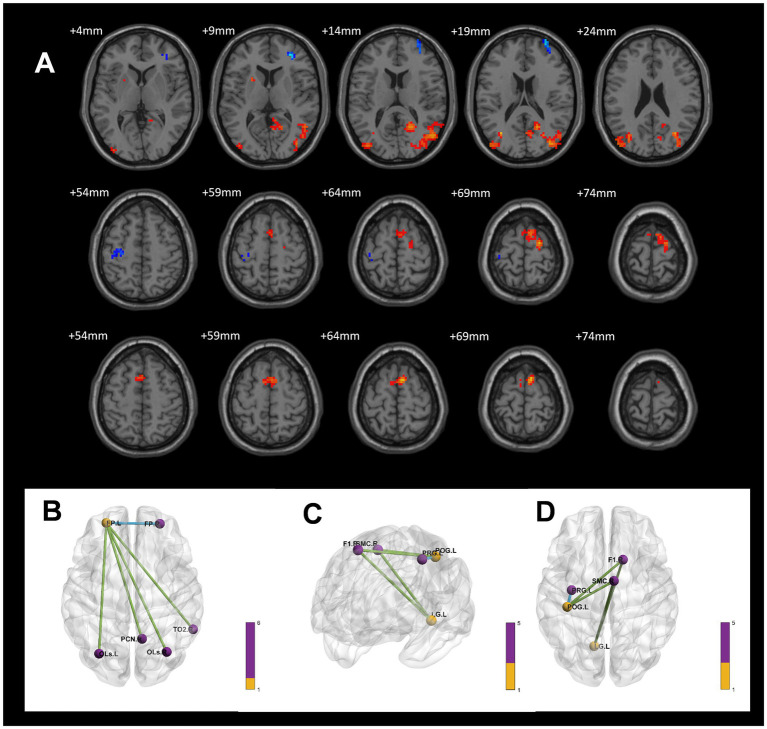
Differences in functional connectivity between the CWP drinking group, and the CWP non-drinking group. **(A)** Compared with the CWP non-drinking group, the left frontal pole had increased strength of connectivity to the bilateral Superior Occipitolateral Cortex, the right Middle Temporal Gyrus, and the right Precuneus Cortex, while the left frontal pole had decreased connectivity to the right Frontal Pole in the CWP drinking group. Both the left Postcentral Gyrus and the left Lingual Gyrus had increased connectivity to the right Juxtaposed Lobular Cortex and the right Superior Frontal Gyrus, while the left Postcentral Gyrus had diminished connectivity to the left Precentral Gyrus (GRF, voxel *p* < 0.005, cluster *p* < 0.05). **(B)** The three-dimensional connectivity maps of the brain in the left Frontal Pole (axial), **(C,D)** The Brain 3D connectivity maps (sagittal, axial) of left Postcentral Gyrus, right Lingual Gyrus. Green edges represent enhanced functional connectivity, blue edges represent weakened functional connectivity, L: left side, R: right side.

Compared to the CWP non-drinking group, the CWP drinking group showed increased connectivity of the left Frontal Pole to the upper part of the bilateral Lateral Occipital Cortex and the right Middle Temporal Gyrus to the right Precuneus Gyrus, while the connectivity of the left Frontal Pole to the right Frontal Pole was decreased. Additionally, the left Postcentral Gyrus and the left Lingual Gyrus exhibited increased connectivity to the right Juxtaglomerular Cortex and the right Superior Frontal Gyrus, but the left Postcentral Gyrus had decreased connectivity to the left Precentral Gyrus (GRF, voxel *p* < 0.005, cluster *p* < 0.05, Figure 5).

**Figure 6:**
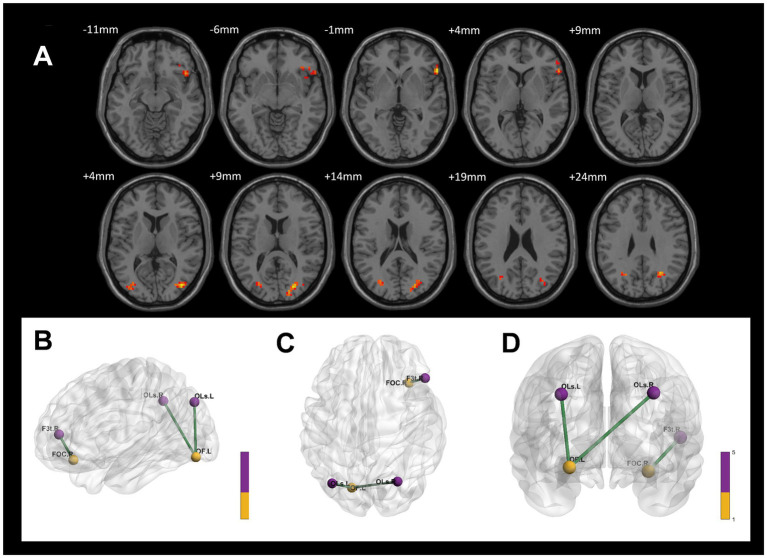
Differences in functional connectivity between the CWP drinking group and the control group. **(A)** The connectivity between the right Orbitofrontal Cortex to the delta of the right Inferior Frontal Gyrus, and the left Occipital Fusiform Gyrus to the bilateral Lateral Occipital Cortex was increased in the CWP drinking group compared to the control group (GRF, voxel *p* < 0.005, cluster *p* < 0.05). **(B–D)** The three-dimensional connectivity maps of the left Frontal Orbital Cortex, right Occipital Fusiform Gyrus, Green edges represent enhanced functional connectivity. L: left side, R: right side.

Compared to the control group, the CWP drinking group demonstrated increased connectivity of the right Orbitofrontal Cortex to the delta of the right Inferior Frontal Gyrus and the left Occipital Fusiform Gyrus to the bilateral Lateral Occipital Cortex (GRF, voxel *p* < 0.005, cluster *p* < 0.05, Figure 6).

**Figure 7:**
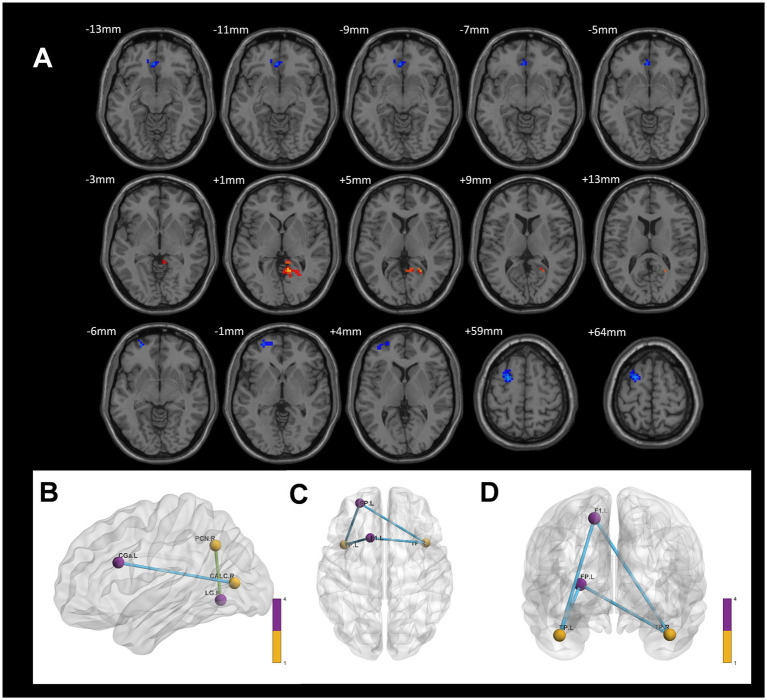
Differences in functional connectivity between the CWP non-drinking group and the control group. **(A)** The functional connectivity of the right Precuneus Cortex to the right Lingual Gyrus was increased in the CWP non-drinking group compared to the control group. The functional connectivity of the right Intracalcarine Cortex to the anterior part of the left Cingulate Gyrus, the bilateral Temporal Pole to the left Superior Frontal Gyrus, and the left Frontal Pole was weakened (GRF, voxel *p* < 0.005, cluster *p* < 0.05). **(B)** The brain 3D connectivity maps of the right Intracalcarine Corte, and the left Precuneus Cortex. **(CD)** The brain 3D connectivity map of Bilateral Temporal Pole. Green edges represent enhanced functional connectivity and blue edges represent reduced functional connectivity. L: left side, R: right side.

In comparison to the control group, the CWP non-drinking group showed increased connectivity of the right Precuneus Cortex to the right Lingual Gyrus. Conversely, decreased connectivity was observed between the right Intracalcarine Cortex and the anterior part of the left Cingulate Gyrus, as well as between the bilateral Temporal Poles and the left Superior Frontal Gyrus and the left Frontal Pole (GRF, voxel *p* < 0.005, cluster *p* < 0.05, Figure 7).

### 3.4 Correlation analysis

Spearman correlation analysis revealed a positive correlation between ALFF values in the right Intracalcarine Cortex and both FEV1 (r = 0.386, *p* = 0.029) and HAMA (r = 0.370, *p* = 0.037) in the CWP drinking group. Additionally, ALFF values in the left Temporal Pole showed a positive correlation with PaO2 (r = 0.369, *p* = 0.038), suggesting that alterations in PaO2 may serve as an important structural basis for overall cognitive decline. The left Postcentral Gyrus ALFF values demonstrated a positive correlation with HAMD (r = 0.383, *p* = 0.030), while the left Occipital Fusiform Gyrus ALFF values exhibited a negative correlation with years of drinking (r = −0.381, *p* = 0.032).

**Figure 8:**
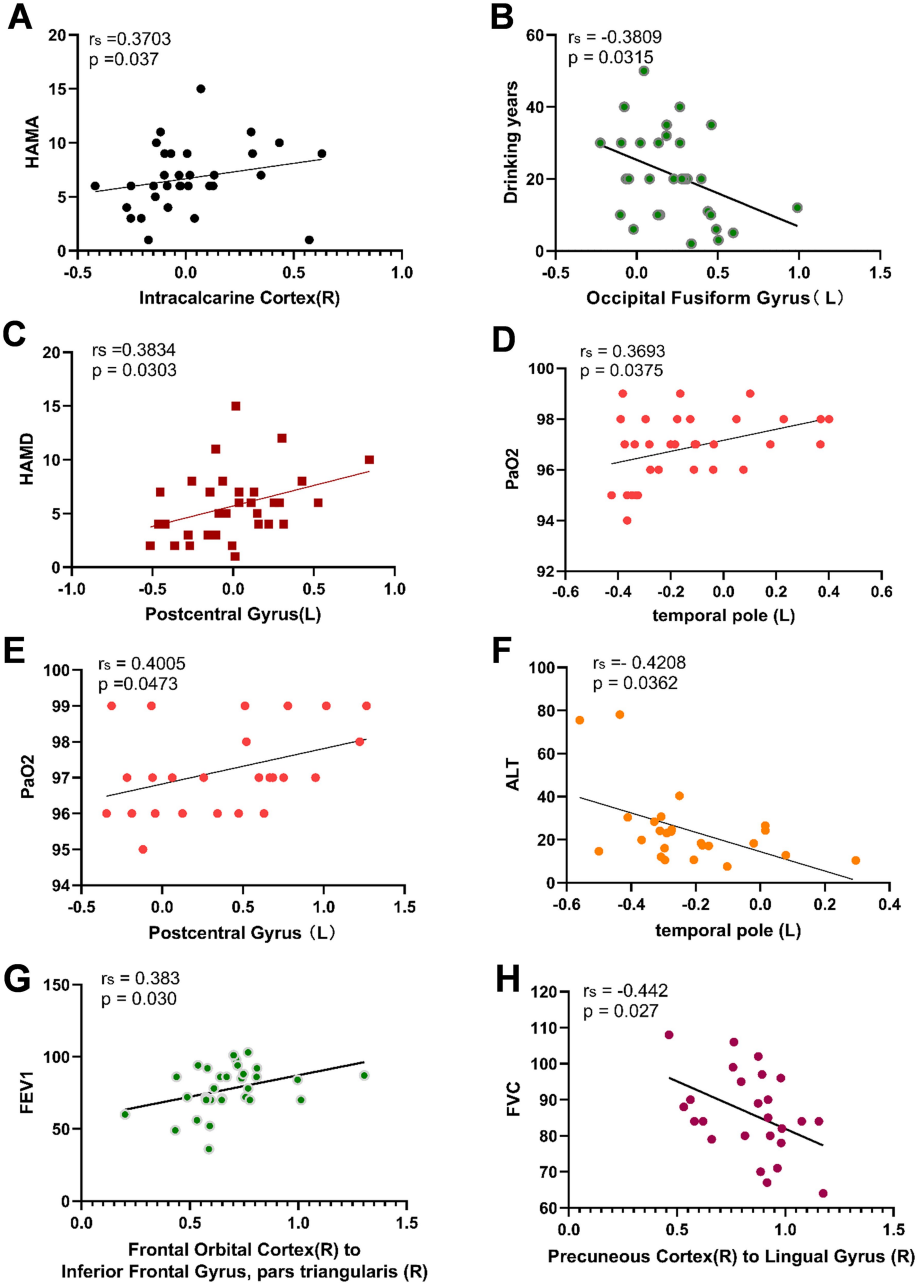
Correlation analysis between abnormal ALFF, FC values and related cognitive clinical indicators in CWP patients. Right Intracalcarine Cortex was positively correlated with HAMA in the CWP drinking group **(A)**, left Occipital Fusiform Gyrus was negatively correlated with the number of drinking years **(B)**, left Postcentral Gyrus ALFF value was positively correlated with HAMD **(C)**, left Temporal Pole was positively correlated with PaO2 **(D)**. In the CWP non-drinking group, the left Posterior Central Gyrus was positively correlated with PaO2 **(E)**, and the left Temporal Pole was negatively correlated with ALT **(F)**. The right Orbitofrontal Cortex to the right Inferior Frontal Gyrus in the CWP drinking group were positively correlated with FEV1 **(G)**. Functional connectivity of the right Precuneus Cortex to the right Lingual Gyrus was significantly negatively correlated with FVC in the CWP non-drinking group **(H)**. The correlation analyses of the CWP non-drinking group were shown in Groups **A-D** for the drinking group, and Groups **E-F** for the non-drinking group. PaO2: partial pressure of blood oxygen, ALT: alanine aminotransferase, HAMA: Hamilton Anxiety Scale, HAMD: Hamilton Depression Scale, FEV1: forceful expiratory volume at the end of 1 s, FVC: forceful lung volume, L: left side, R: right side.

In the CWP non-drinking group, the left Postcentral Gyrus ALFF values showed a significant positive correlation with PaO2 (r = 0.401, *p* = 0.047), and a significant negative correlation was observed between left Temporal Pole ALFF values and ALT (r = −0.421, *p* = 0.036, Figure 8). However, no significant correlations were found between the remaining cognitive-related clinical assessment indicators and the abnormal brain regions (*p* > 0.05, Table 3).

**Table 3:** Spearman correlation between ALFF values in abnormal brain regions and clinical indicators.

Characteristic	Postcentral Gyrus (L)		Intracalcarine Cortex (R)		Temporal pole (L)	
	Nondrinking groups	Drinking groups	Nondrinking groups	Drinking groups	Nondrinking groups	Drinking groups
Education	r = 0.322, *p* = 0.115	r = −0.245, *p* = 0.177	r = 0.237, *p* = 0.255	r = 0.153, *p* = 0.404	r = −0.132, *p* = 0.529	r = 0.235, *p* = 0.195
Working years	r = −0.041, *p* = 0.843	r = −0.016*, p* = 0.932	r = 0.013*, p* = 0.952	r = −0.231*, p* = 0.203	r = −0.073*, p* = 0.731	r = 0.008*, p* = 0.963
Drinking years		r = 0.114*, p* = 0.533		r = 0.304*, p* = 0.091		r = 0.166*, p* = 0.365
AC/tael		r = 0.235*, p* = 0.195		r = 0.233*, p* = 0.200		r = 0.099*, p* = 0.589
ALT	r = 0.353*, p* = 0.083	r = 0.015*, p* = 0.933	r = 0.222*, p* = 0.286	r = −0.215*, p* = 0.237	r = −0.421, *p* = 0.036	r = −0.261*, p* = 0.150
FVC	r = −0.065*, p* = 0.758	r = 0.338*, p* = 0.059	r = −0.371*, p* = 0.068	r = 0.246*, p* = 0.175	r = −0.101*, p* = 0.630	r = −0.090*, p* = 0.623
FEV1	r = −0.020*, p* = 0.924	r = 0.205*, p* = 0.261	r = −0.284*, p* = 0.169	r = 0.386*, p* = 0.029	r = −0.167*, p* = 0.424	r = 0.066*, p* = 0.721
FEV1/FVC	r = −0.034*, p* = 0.874	r = −0.152*, p* = 0.406	r = −0.276*, p* = 0.181	r = 0.125*, p* = 0.497	r = 0.083*, p* = 0.694	r = 0.198*, p* = 0.277
PaO2	r = 0.410*, p* = 0.047	r = −0.323*, p* = 0.072	r = 0.249*, p* = 0.230	r = 0.083*, p* = 0.653	r = −0.376*, p* = 0.064	r = 0.369*, p* = 0.038
MMSE	r = 0.312*, p* = 0.130	r = 0.144*, p* = 0.433	r = 0.215*, p* = 0.303	r = −0.171*, p* = 0.349	r = 0.195*, p* = 0.351	r = −0.055*, p* = 0.763
MoCA	r = 0.030*, p* = 0.888	r = 0.199*, p* = 0.275	r = 0.046*, p* = 0.828	r = 0.008*, p* = 0.965	r = −0.028*, p* = 0.895	r = −0.067*, p* = 0.715
SCD-Q	r = −0.023*, p* = 0.912	r = 0.288*, p* = 0.109	r = −0.183*, p* = 0.686	r = −0.074*, p* = 0.686	r = −0.149*, p* = 0.477	r = 0.066*, p* = 0.719
HAMD	r = 0.061*, p* = 0.774	r = 0.383*, p* = 0.030	r = −0.152*, p* = 0.468	r = 0.281*, p* = 0.120	r = −0.070*, p* = 0.741	r = −0.159*, p* = 0.385
HAMA	r = −0.209*, p* = 0.316	r = 0.167*, p* = 0.362	r = −0.214*, p* = 0.304	r = 0.370*, p* = 0.037	r-0.012*, p* = 0.956	r = 0.157*, p* = 0.390
GDS	r = −0.218*, p* = 0.296	r = 0.375*, p* = 0.034	r = −0.300*, p* = 0.145	r = 0.091*, p* = 0.622	r = 0.086*, p* = 0.681	r = 0.050*, p* = 0.786
CDT	r = 0.067*, p* = 0.749	r = −0.097*, p* = 0.599	r = 0.210*, p* = 0.315	r = −0.258*, p* = 0.154	r = −0.069*, p* = 0.743	r-0.176*, p* = 0.335

AC/tael, Alcohol Consumption per tael; MMSE, Minimum Mental State Examination; MoCA, Montreal Cognitive Assessment; SCD-Q, Subjective Cognitive Decline questionnaire; HAMD, Hamilton Depression Scale; HAMA, Hamilton Anxiety Scale; GDS, geriatric depression scale; CDT, Clock Drawing Test; L: Left, R: Right.

Functional connectivity of the right Orbitofrontal Cortex to the right Inferior Frontal Gyrus was positively correlated with FEV1 in the CWP drinking group (r = 0.383, *p* = 0.030). Conversely, a negative correlation was observed between the functional connectivity of the right Precuneus Cortex to the right Lingual Gyrus and FVC in the CWP non-drinking group (r = −0.442, *p* = 0.027, Figure 8). These findings suggest that diminished lung function indices may play a role in the alterations in functional connectivity observed in CWP patients. However, no significant correlations were found between cognitive-related clinical assessment indices and the functional connectivity of abnormal brain regions (*p* > 0.05, Table 4).

**Table 4:** Spearman correlation between FC values of abnormal brain regions and clinical indicators.

Characteristic	Frontal Pole (L) to Frontal Pole (R)		Frontal Orbital Cortex (R) to Inferior Frontal Gyrus, pars triangularis (R)	Precuneus Cortex (R) to Lingual Gyrus (R)
	Drinking groups	Nondrinking groups	Drinking groups	Nondrinking groups
Education	r = −0.283*, p* = 0.117	r = 0.187*, p* = 0.370	r = −0.045*, p* = 0.802	r = 0.361*, p* = 0.076
Working years	r = 0.069*, p* = 0.709	r = −0.048*, p* = 0.819	r = 0.017*, p* = 0.925	r = −0.030*, p* = 0.888
Drinking years	r = −0.176*, p* = 0.336		r = −0.193*, p* = 0.291	
AC/tael	r = 0.011*, p* = 0.950		r = −0.074*, p* = 0.685	
ALT	r = −0.098*, p* = 0.593	r = 0.197*, p* = 0.345	r = 0.163*, p* = 0.374	r = 0.144*, p* = 0.493
FVC	r = 0.116*, p* = 0.527	r = −0.292*, p* = 0.156	r = 0.294*, p* = 0.103	r = −0.442*, p* = 0.027
FEV1	r = 0.069*, p* = 0.709	r = −0.155*, p* = 0.458	r = 0.383*, p* = 0.030	r = −0.290*, p* = 0.160
FEV1/FVC	r = −0.141*, p* = 0441	r = 0.039*, p* = 0.855	r = 0.214*, p* = 0.241	r = −0.251*, p* = 0.226
PaO2	r = −0.029*, p* = 0.876	r = 0.322*, p* = 0.116	r = −0.056*, p* = 0.760	r = 0.210*, p* = 0.313
MMSE	r = 0.097*, p* = 0.600	r = −0.017*, p* = 0.932	r = 0.212*, p* = 0.245	r = 0.229*, p* = 0.272
MoCA	r = −0.044*, p* = 0.812	r = −0.044*, p* = 0835	r = 0.290*, p* = 0.108	r = 0.206*, p* = 0.323
SCD-Q	r = 0.281*, p* = 0.120	r = 0.000*, p* > 0.999	r = 0.193*, p* = 0.291	r = −0.119*, p* = 0.572
HAMD	r = −0.049*, p* = 0.790	r = 0.084*, p* = 0.687	r = −0.179*, p* = 0.327	r = −0.164*, p* = 0.433
HAMA	r = 0.128*, p* = 0.487	r = 0.082*, p* = 0.698	r = −0.119*, p* = 0.518	r—0.326*, p* = 0.112
GDS	r = 0.114*, p* = 0.535	r = −0.092*, p* = 0.661	r = 0.038*, p* = 0.835	r = −0.275*, p* = 0.183
CDT	r = 0.002*, p* = 0.990	r = 0.065*, p* = 0.756	r = 0.090*, p* = 0.623	r = 0.337*, p* = 0.099

AC/tael, Alcohol Consumption per tael; MMSE, Minimum Mental State Examination; MoCA, Montreal Cognitive Assessment; SCD-Q, Subjective Cognitive Decline questionnaire; HAMD, Hamilton Depression Scale; HAMA, Hamilton Anxiety Scale; GDS, geriatric depression scale; CDT, Clock Drawing Test; L: Left, R: Right.

## 4 Discussion

The results of this study demonstrated that pulmonary function indices (FVC, FEV1, FEV1/FVC) were higher in the CWP drinking group compared to the non-drinking group, suggesting that alcohol consumption may have a positive effect on the improvement of ventilatory function. However, the change in PaO2 was not significantly different between the two groups. The MMSE scores were comparable between the two groups, while the MoCA, SCD-Q, HAMA, HAMD, and GDS scores were higher in the drinking group than in the non-drinking group, indicating greater cognitive impairment in the non-drinking group of the CWP, which was manifested in the scale as diminished ability in executive function and delayed recall. On the other hand, alcohol may have less effect on emotional functioning in depression and anxiety. In contrast, scores on the CDT were lower in the drinking group, suggesting a correlation with visuospatial and recall reproduction abilities.

In the present study, we found increased overall ALFF signal in the right Precuneus Cortex, right Intracalcarine Cortex, Bilateral Orbitofrontal Cortex in a one-way ANOVA analysis between multiple groups of CWP. The left Orbitofrontal Cortex had increased connectivity to the right Precuneus Cortex, the left Frontal Pole had increased connectivity to the bilateral Precuneus Cortex, and the right Precuneus Cortex had increased connectivity to the right Lingual Gyrus. The Precuneus Cortex is thought to be more closely associated with the default mode network (DMN), an interconnected and highly correlated neural network (Menon, 2023) that is active at rest and has been found to be highly correlated with psychiatric disorders such as AD, Depression, and Autism (Giorgio et al., 2024; Chou et al., 2023; Yang et al., 2023). In the DMN network, the Precuneus Cortex is associated with memory perception, attention, and sensorimotor information integration (Dadario and Sughrue, 2023), and diseases can damage the Precuneus Cortex to varying degrees, resulting in dysfunction or even organic changes. It has been found that under the influence of negative emotions, Precuneus Cortex can show negative activation in fMRI (Moe et al., 2023). Run et al. also found that subjects had reduced ALFF signals in the left Precuneus Cortex under alcohol induction (Liu et al., 2018). On the other hand, cortical thickness of the Precuneus Cortex can be significantly reduced in the presence of epilepsy (Du et al., 2023). In the above studies, either in the state of negative DMN activation or under the influence of chronic alcohol, the Precuneus Cortex showed signal reduction in fMRI. However, the results of the multiple group comparison in the present study showed increased ALFF signal in the Precuneus Cortex and increased functional connectivity between the Precuneus to several brain regions, suggesting that the DMN in CWP patients with alcoholism were persistently active, reflecting the activation of memory and attention networks in patients with CWP. Previous studies have shown that the precuneus is functionally heterogeneous, exhibiting both anterior region’s self-referential thinking and posterior region’s episodic memory and visuospatial intention (He et al., 2025), The Complex function of precuneus may represent a lesser degree of damage to early CWP. On the other hand. This may be due to early cognitive impairment, leading the Precuneus Cortex to maintain general neural activity through compensatory mechanisms. This performance contributes to our enhanced understanding of abnormal brain activity in CWP patients under the influence of alcoholism.

In contrast to the non-drinking group, the left Middle Frontal Gyrus (MFG) ALFF signaling was reduced in the drinking group, whereas there was no significant alteration in functional connectivity in the left MFG. The primary functions of the MFG include the head-eye movement and writing centers (Briggs et al., 2021), while its memory-related functions are mainly associated with working memory, attention, and language-related processing. Current research on the MFG has primarily focused on psychiatric disorders such as subclinical depression, anxiety-type depression, and schizophrenia (Zhao et al., 2019; Li et al., 2023). Previous studies have reported brain abnormalities in patients with amnestic cognitive impairment (aMCI) (Wu et al., 2023), with alterations in ALFF signaling mainly observed in the right Superior Frontal Gyrus and MFG of patients with aMCI (Liu et al., 2022). These findings align with the results of the present study, which reported reduced ALFF signals in the left MFG in a chronic alcohol environment. Under the indulge of alcohol, complex decision-making and working memory functions involving the MFG are thought to be impaired, resulting in reduced spontaneous neural activity, thus contributing to cognitive decline. These findings provide evidence supporting the association between alcoholism and cognitive dysfunction in patients.

Compared to the control group, the CWP drinking group exhibited increased ALFF signals in the Orbitofrontal Cortex (OFC) bilaterally, as well as increased connectivity between the right OFC and the delta of the right Inferior Frontal Gyrus. According to the Brodmann partitioning system, the OFC is located in area 47 (BA47), situated in the middle of the Ventral Frontal Lobe bilaterally, at the junction between the Prefrontal Cortex and the Limbic system. It primarily manages the expression of the reward system and emotional responses (Ruge et al., 2025). Previous studies have identified increased functional connectivity of the OFC to the Precuneus Cortex and Posterior Cingulate Cortex in depressed patients (Karolewicz et al., 2010; Zhang et al., 2024), suggesting that early connectivity changes are associated with stress-related cognitive deficits and mood dysregulation. Alcohol use disorder has also been linked to altered functional connectivity in cortico-amygdala-striatal circuits (Nimitvilai-Roberts et al., 2025; Radoman et al., 2024), with studies reporting increased ALFF signals and functional connectivity in the OFC. This suggests that the craving for alcohol-related cues and the functioning of the OFC’s reward system may influence the onset and progression of AUD. The findings of this study support this evidence, Under the dual influence of CWP and Alcoholism, the activation of reward system is dominant, which indicating that the OFC could serve as a biomarker for analyzing neuroimaging changes in CWP patients with alcoholism.

On the other hand, compared to the control group, the CWP non-drinking group showed reduced ALFF signaling in the bilateral Temporal Poles and reduced functional connectivity of the bilateral Temporal Poles to the left Superior Frontal Gyrus and the left Frontal Pole. According to the Brodmann partitioning system, the rostral portion of the anterior temporal lobe is referred to as the Temporal Pole (BA38), which primarily manages memory storage and the expression of affective functions (Mesulam, 2023). It is specifically involved in cognitive processes such as autobiographical memory, complex visual processing, naming, and socio-emotional processing (Herlin et al., 2021). Under the influence of neurodegenerative pathologies, dysfunction in the Temporal Pole can lead to neurological disorders such as behavioral dementia, primary progressive aphasia, epilepsy, and other conditions (Gosnell et al., 2020; Herlin et al., 2020). In the present study, ALFF signaling and functional connectivity in the bilateral Temporal Poles were significantly reduced in CWP patients compared to controls. This reduction may be commonly attributed to chronic hypoxia-induced alterations in spontaneous neural activity. Previous studies have shown reduced BOLD signaling in the visual cortex under hypoxic conditions, which supports this explanation (Arngrim et al., 2019). On the other hand, Setton et al. (2022) found that functional connectivity in both the anterior and posterior hippocampus and Temporal Poles decreases with aging. However, in the present study, the age difference between the groups was not statistically significant, suggesting that the observed alterations in neural activity in the Temporal Poles are not directly attributable to age. AD is the most common neurodegenerative disease in the elderly, is characterized by region-specific nonlinear atrophy throughout the brain as it progresses from MCI to AD stages. This atrophy is most likely to occur in the superior Temporal Pole, caudate nucleus, and hippocampus (Wei et al., 2023). The findings of the present study, which reported reduced ALFF signaling and functional connectivity in the Temporal Pole bilaterally, align with this interpretation and provide further evidence of the vulnerability of the Temporal Pole to neurodegenerative processes.

The partial pressure of oxygen (PaO2) is an indicator of the tension of O2 physically dissolved in the blood at room temperature and pressure (Attaway et al., 2024). In the correlation analysis of the present study, ALFF values in the left Temporal Pole and the left Postcentral Gyrus showed a significant positive correlation with PaO2. This is hypothesized to be due to the clogging of alveoli and distal fine bronchioles by coal dust microparticles in the lungs of patients with CWP, which leads to a reduction in the oxygen content in the blood (Fan et al., 2022). Previous studies have supported the presence of altered nocturnal hypoxemia in patients with interstitial lung fibrosis (Myall et al., 2023). However, in this study, the PaO2 levels in CWP patients were above the lower limit of normal. This may be because the experimental group consisted of patients in the early stages of CWP, their imaging features are characterized by multiple diffuse micronodules in both lungs, partial blockage of alveoli, and impaired respiratory exchange thus affects brain function and metabolism. Where the body compensates to maintain normal PaO2 levels, suggesting that the effect of coal dust microparticles on alveoli and distal fine bronchioles in the early stage is relatively small.

This study has several limitations. First, the sample size of the CWP subgroup was relatively small, and future studies will expand the sample size to ensure a more accurate presentation of the results. Second, this study was cross-sectional and did not analyze brain function abnormalities over the course of CWP progression in a longitudinal manner. Lastly, the study did not account for the effects of long-term maintenance medications, other than sedative-hypnotic medications, in patients with CWP. Future follow-up studies will aim to comprehensively capture possible influencing factors and include them in the analysis of covariates, reducing the likelihood of these factors affecting scale score results and differences in brain area function.

## 5 Conclusion

Our study demonstrates that there are abnormalities in brain regions of spontaneous neural activity in CWP patients with alcoholism. The differences in ALFF and FC in the left Orbitofrontal Cortex, the right Intracalcarine Cortex, and the right Precuneus Cortex are closely associated with cognition-related clinical indicators. These brain regions with significant differences may serve as potential biomarkers for cognitive function assessment in CWP patients with alcoholism. The findings of this study provide valuable reference points for neurological research based on neuroimaging of cognitive impairment.

## Data Availability

The raw data supporting the conclusions of this article will be made available by the authors, without undue reservation.

## Footnotes

^1^ www.mathworks.com/products/new_products/release2023a.html

^2^ www.nitrc.org/projects/bnv/
